# 

**Published:** 1918-05

**Authors:** 


### ANNOUNCEMENTS

The next meeting of the National Association of Dental Examiners will be held in Chicago, Ill., August 5 and 6, at the Auditorium Hotel. For further information address the secretary, Dr. J. A. West, 417 Utica Bldg., Des Moines, Iowa.

The eighteenth annual meeting of the American Society of Orthodontists will be held August 1, 2 and 3, at the Edgewater Beach Hotel, Chicago, Ill. This will be an excellent meeting. It is advisable to make your reservations early.

*Michigan State Board.* The next meeting of the Michigan State Board of Dental Examiners will be held at the dental college, University of Michigan, Ann Arbor, Mich., June 17 to 22, inclusive. Applications and other information may be obtained by addressing B. S. Sutherland, Secretary, Owosso, Mich.

*Kentucky State Dental Association.* The next annual meeting of the Kentucky State Dental Association will be held at Lexington, Ky., June 13, 14, 15, 1918. An "amalgam program of special interest." Address all correspondence to Dr. W. M. Randall, Secretary, Louisville, Ky.

*West Virginia State Board.* The annual examination of candidates for license to practice in the state of West Virginia will be held at Parkersburg, W. Va., June 25, 26 and 27, 1918. Blanks and information will be furnished on application to the undersigned. H. H. Smallridge, Secretary.

### PREPAREDNESS LEAGUE NEWS AND NOTES

### COMMUNICATION FROM THE PRESIDENT, J. W. BEACH

It is important to keep before the minds of all members, the fact that we should devote our energies almost entirely to men qualified for general military service. These are the men who will do the actual fighting and who must live in the trenches, and who, consequently, are more subject to sickness and infection.

*Dental Requirements and Emergency Work.* There seems to be a misunderstanding on the part of some as to what the dental requirements are to fit a man for "general military service." He must have six opposing incisors and six opposing masticating teeth (either bicuspids or molars). These bicuspids or molars may be all on one side or part on each side, but there must be at least three above and three below, each of which must touch some tooth on the opposite jaw.

The most important thing to do for these men is to rid their mouths of bad roots and infected teeth, or any tooth having a history of periodical abscess.

After the mouths are freed from infection and the gums healed, plates can be made by members of the league who desire to do so, or they can be made after reaching the cantonments, if the officer in charge deems it necessary.

The mouths, however, must *first* be made free from infection. Then, if possible, any large cavities in the remaining teeth should be filled and the teeth cleaned.

The lack of the minimum number of teeth (six and six) does not excuse any man from *military* service, but only from "general military service."

The man who has less teeth than the minimum required for *general* military service can be accepted (if otherwise fit) for limited military service, even if he has *no* teeth.

These men for limited military service will cook food, drive wagons and auto trucks, carry supplies, work in factories, shops or storehouses, etc.

*Our Next Move.* We do not want members of the league to get the idea that the great drive now going on to help make our national army dentally fit is the sole object of our organization. It is but one of a series of our activities. The development of our dental motor car is a signal illustration of the great possibilities before us.

Those of us who are not commissioned and continue in civil practice must devote our energies to prepare to give expert services to our soldiers who have been injured in battle. We will find ourselves in need of all the skill we can summon, therefore, I would impress upon our members the great advantage of forming sectional units of the league throughout the whole country for the study of war oral and dental surgery. We are proud to say that our dental reserve is overfilled and service in that direction is amply provided for. However, if later on more are needed, the study course will the better prepare us. I, therefore, strongly urge the promotion of this object and point with pride to the splendid record the league has already made in that direction. More than one hundred sectional units have given such a course which has been the means of assisting several hundred to pass examinations to the reserve corps, as well as promoting their advancement to higher commissions and positions in the service.

*Lecture and Slides.* The league has prepared a synopsis lecture accompanied by slides showing cases of plastic and oral surgery before and after treatment; a series covering our free dental activities and a third, showing our new dental motor car.

Twenty-five sets have been distributed to different state directors for use by sectional units of the league and all other societies. We urge their use at state society meetings. The laity, also, will be interested. Assignments may be made by your state director or through the office of the president, 131 Allen Street, Buffalo, N. Y.

*Important Notice.* It has come to the attention of the president of the league that a pamphlet is being circulated advising our members to charge selective service men who are able to pay, for the service necessary to make them dentally fit.

This pamphlet is *spurious* and the originators deserve no better treatment than internment during the war as cohorts of the kaiser. It is but another vain attempt to abort the principles upon which the league is founded and which will live for ages after the perpetrators of this unAmerican and despicable subterfuge have become naught but a blotch of mold upon our fair soil and the unfairly acquired gain has been dissipated without benefit to themselves or their families.

I trust all loyal dentists will exert themselves to give this stigma the lie by strictly adhering to the only principle that can make our organization live and be of vital importance to humanity and our country in this great crisis.

*League Membership.* The league has a membership of 13,600 to date. This number should be increased to 30,000 members at once. Ask your county directors to endeavor to get every member to enlist at least one more member.

*Operations Performed.* The United States now has more than 1,500,000 men under arms, and up to date we have records of work done by the league, of approximately 200,000 operations, but if each had done his part, this number would have been doubled. Some individual members have performed as many as 150 operations; others (new members), none at all, as they have not yet had the opportunity.

Our organization should perform 1,000,000 operations for the men of the next draft.

